# Disitamab vedotin (RC48) long-term regimen in a post-nephroureterectomy patient with metastases: a case report

**DOI:** 10.3389/fonc.2024.1419882

**Published:** 2024-09-13

**Authors:** Weiping Li, Suoshi Jing, Bo Zhao, Wei Jiang, Bin Zhang

**Affiliations:** ^1^ Department of Urology, Lanzhou University First Affiliated Hospital, Lanzhou, Gansu, China; ^2^ Department of Urology, Joint Logistic Support Force 940th Hospital of Chinese People's Liberation Army (PLA), Lanzhou, Gansu, China; ^3^ Convalescent Section First of Convalescent Zone Second, Air Force Hangzhou Secret Service Rehabilitation Center, Hangzhou, Zhejiang, China

**Keywords:** immunotherapy, disitamab vedotin (RC48), metastasis, upper tract urothelial carcinoma, GC

## Abstract

**Background:**

For patients with metastatic upper tract urothelial carcinoma (UTUC), the preferred first-line treatment is platinum-based chemotherapy. Immunotherapy can be considered a subsequent treatment if the chemotherapy is ineffective or the disease progresses. However, how should treatment be administered if immunotherapy is useless and the patient cannot take chemotherapy? Especially in patients who have metastasized after radical nephroureterectomy (RNU).

**Case presentation:**

A 68-year-old woman was admitted to the hospital due to left flank pain for two weeks and was diagnosed with metastatic UTUC after RNU. The patient underwent systemic chemotherapy with gemcitabine and cisplatin (GC), as well as reduced-dose GC, but developed myelosuppression. Immunotherapy was ineffective, so Disitamab vedotin (RC48) was chosen.

**Results:**

Disitamab vedotin (RC48) monotherapy was administered for 13 cycles, during which thyroid function remained normal, the patient responded well to the treatment, and the disease was controlled well. In the subsequent two years of follow-up, there was no disease recurrence.

**Conclusion:**

The long-term treatment regimen with RC48 is feasible for metastatic UTUC after RNU, and RC48 monotherapy is suitable as first-line therapy for selected patients.

## Introduction

Upper tract urothelial carcinoma (UTUC) arising in the renal pelvis or ureter accounts for only 5–10% of urothelial carcinomas (1). It is an aggressive malignancy that is challenging to treat. Over the past decade, its incidence has been rising, likely due to an aging population and improved detection methods, but survival rates have been minimal. In China, however, the incidence rate ranges from 9.3% to 29.9%, with a higher prevalence in females (2). When diagnosed, about two-thirds of patients with UTUC have invasive disease (3). 7-17% of UTUC cases are concurrent with bladder cancer (2).

In localized low-risk UTUC, kidney-sparing surgery is a preferred approach, as survival is similar to that after radical nephroureterectomy (RNU) (4); RNU is the standard treatment for high-risk UTUC, regardless of tumor location (3). For patients with metastatic UTUC, cisplatin-based combination chemotherapy is the standard treatment (1, 5). If the chemotherapy is ineffective or the disease progresses, immunotherapy can be considered a subsequent treatment, and antibody-drug conjugates (ADC) can be used as a third-line setting (1). Nevertheless, there are no current recommendations for treating patients with metastatic UTUC following RNU. Specifically, how should patients be treated when they are not responding to immunotherapy and cannot take platinum-based chemotherapy?

This study details our practical case-specific therapy experience. After RNU, the patient with concurrent bladder cancer and UTUC experienced metastases. The patient’s condition was well-controlled, and metastatic cancer vanished following treatment, thanks to the long-term monotherapy with Disitamab vedotin (RC48) that we selected. RC48 is an innovative anti-HER2 ADC that combines hertuzumab (a novel anti-HER2 mAB) with monomethyl auristatin E by a cleavable linker. RC48 has been approved for the treatment of cancer patients who have undergone at least two rounds of systemic chemotherapy and have HER2+ (immunohistochemistry, IHC, 2+/3+) locally advanced or metastatic gastric carcinoma, gastric or gastroesophageal junction carcinoma, and urothelial carcinoma (6, 7).

The treatment experience, in this case, indicates that a long-term treatment regimen with RC48 monotherapy is feasible for metastatic UTUC after RNU and suitable as first-line therapy for selected patients.

## Case presentation

### Chief complaints

A 68-year-old woman was admitted to the hospital due to left flank pain for two weeks.

### Past medical history

The patient was diagnosed with left-sided high-risk UTUC (T1N0M0) complicated with bladder cancer (as shown in **Supplementary Figure S1**) and underwent robotic-assisted RNU and partial cystectomy seven months ago. Intravesical instillation of pirarubicin was given 20 days after surgery, once a week, six times, and once a month for four months. The histological examination revealed a low-grade, non-invasive urothelial carcinoma (as shown in **Supplementary Figure S2**). The patient reported no history of hypertension, trauma, drug or food allergies, radiation exposure, or poison exposure in the family.

### Laboratory examinations (January 19, 2021)

The estimated glomerular filtration rate (eGFR) was 69.94 mL/min/1.73 m^2^. White blood cell count (WBC) was 3.95 (normal range: 3.69-9.16) ×10^9^/L; neutrophil count was 2.57 (normal range: 2.00-7.00) ×10^9^/L; red blood cell count (RBC) was 4.30 (normal range: 3.68-5.13) ×10^12^/L. Serum urea (UREA) and creatinine (CRE) levels were normal (as listed in **Supplementary Table S1**).

### Imaging examinations and pathology result

**Figure 1:**
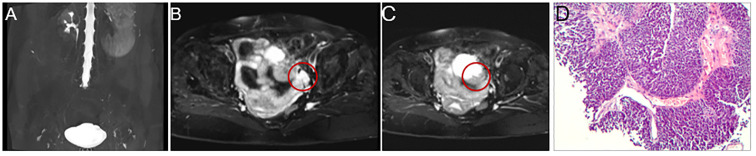
Magnetic resonance (MR) urography and pathology result. MR urography shows the absence of the left kidney and ureter **(A)**, and there is a 2 × 3 cm mass in the left posterior portion of the bladder that exhibits iso-intense T1 and extended T2 signals and a strong signal on diffusion-weighted imaging, all of which point to metastasis (**B**, indicated by the red circle). Additionally, the bladder is well distended, with thickening of the anterior and left lateral walls, reaching a maximum thickness of about 1 cm. It shows isointense T1 and slightly prolonged T2 signals. It exhibits high signal intensity on diffusion-weighted imaging, suggesting tumor recurrence (**C**, indicated by the red circle). The needle biopsy’s pathology result revealed invasive urothelial carcinoma, with the malignant tissue exhibiting notable atypia, varied cell size, and a nest-like distribution **(D)**.

Magnetic resonance (MR) urography revealed the absence of the left kidney and ureter (**Figure 1A**) and a mass of about 2×3 cm in the left posterior aspect of the bladder. The mass exhibited iso-intense T1, prolonged T2 signals, and a high signal on diffusion-weighted imaging, indicating metastasis (**Figure 1B**). The bladder's anterior and left lateral walls were thickened, the T1 signal was equally strong, and the T2 signal was slightly extended. Diffusion-weighted imaging shows high signal intensity, suggesting tumor recurrence (**Figure 1C**). Subsequently, a biopsy of the mass performed under ultrasound guidance confirmed the presence of invasive urothelial carcinoma (**Figure 1D**).

### Final diagnosis

The patient has been diagnosed with metastatic UTUC based on the previously provided information.

### Treatment

#### Gemcitabine and cisplatin chemotherapy

**Figure 2:**
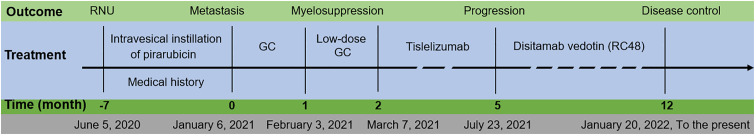
Patient’s overview. The duration of treatment and the evaluation results after the administration of medication. RNU, radical nephroureterectomy; GC, gemcitabine and cisplatin.

The patient’s treatment course is depicted in **Figure 2**. The patient received systemic chemotherapy consisting of gemcitabine (1,800 mg/m^2^ on days 1 and 8) and cisplatin (120 mg/m^2^ on day 2) in a 21-day cycle. The dosages were based on the patient’s body surface area, which was calculated to be 1.711 m^2^.

After one cycle of chemotherapy (February 3, 2021), the patient experienced myelosuppression with low white blood cell count (1.33 ×10^9^/L), neutrophil count (0.81 ×10^9^/L), and platelet count (22 ×10^9^/L), UREA was 5.20(2.4-8.20)mmol/L, CRE was 84.0 (35.0-97.0) μmol/L (as listed in **Supplementary Table S1**). Due to the myelosuppression, chemotherapy was paused, subcutaneous injection of recombinant human granulocyte colony-stimulating factor (150 μg) was administered, and prophylactic treatment against infection was provided for five days and, all blood test results returned to normal levels. Drug discontinuation After discontinuing the chemotherapy drugs for five days and re-examining the patient, all blood test results returned to normal levels (see **Supplementary Table S1**).

#### Low-dose GC chemotherapy

Following that, a reduced dosage of GC (low-dose GC) chemotherapy was administered, consisting of gemcitabine at 1,400 mg/m^2^ and cisplatin at 90 mg/m^2^. After one cycle, the WBC and neutrophil counts decreased again (**Supplementary Table S1**).

#### Immunotherapy

After only two cycles of chemotherapy, the patient experienced grade 3 adverse events, including myelosuppression and gastrointestinal reactions such as nausea and vomiting, indicating that the patient could not tolerate chemotherapy.

**Figure 3:**
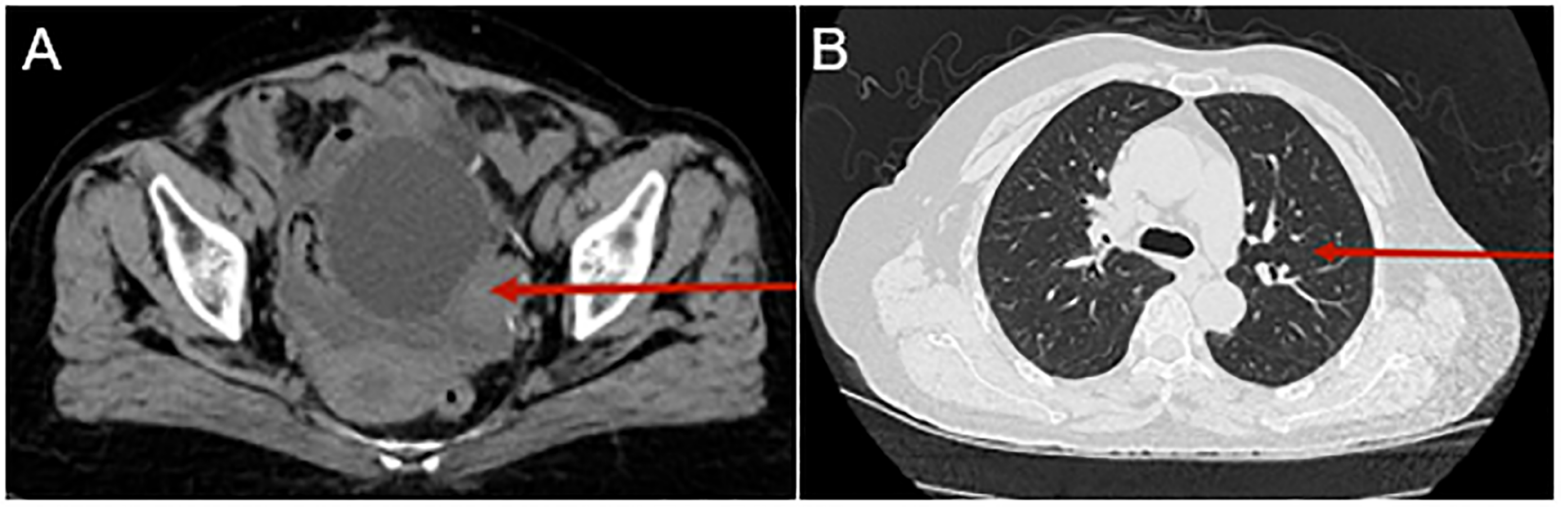
Computed tomography (CT) urography and chest CT following treatment with Tislelizumab. CT urography reveals a mass in the left posterior aspect of the bladder, which has grown since the last scan. The mass is indistinctly bordered by the left posterior wall of the bladder and the left adnexa [as indicated by the red arrow, **(A)**]. The chest CT scan also shows several little nodules in the middle and upper lobes [as shown by the red arrow, **(B)**].

Therefore, immunotherapy using Tislelizumab, a programmed death receptor-1 (PD-1) inhibitor, was switched. It is administered as an intravenous infusion at 200 mg every 21 days. After four cycles of this treatment, computed tomography (CT) results indicated disease progression (see **Figure 3**).

#### Antibody-drug conjugates monotherapy

Considering the over-expression of human epidermal growth factor receptor 2 (HER2) (IHC 3+), RC48 was administered intravenously at a 2.5 mg/kg dose every 14 days.

Assessment following three courses: CT urography shows that the metastatic tumor in the left posterior bladder wall has decreased; Chest CT shows bilateral pleural effusions, with a large volume on the right side, and incomplete expansion of the right lung lobe (**Supplementary Figure S3**). Close thoracic drainage was performed (right side), and after the pleural effusion was fully drained, the drainage tube was removed. Subsequent follow-up did not reveal a recurrence of pleural effusion.

**Figure 4:**
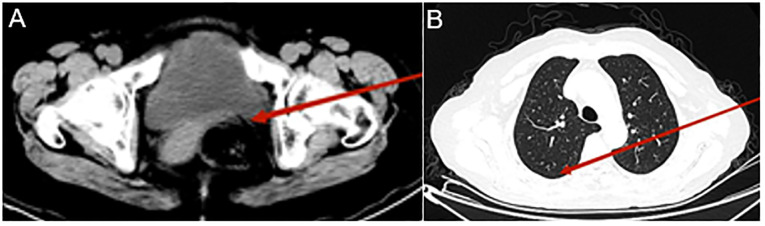
Computed tomography (CT) urography and chest CT after eight Disitamab vedotin (RC48) treatment cycles. CT urography reveals a significantly reduced mass in the left posterior bladder (indicated by the red arrow, **A**); Chest CT shows bilateral pleural effusion absorption, the right lung lobe is fully re-expanded, and the number of lung nodules has decreased (indicated by the red arrow, **B**).

After five treatment cycles, the patient underwent re-evaluation, reporting no left flank pain, nausea, or vomiting. CT examination revealed that the metastatic lesions had disappeared, lung nodules had resolved, and pleural effusion had been absorbed (**Figure 4**).

RC48 was administered for 13 cycles, during which thyroid function remained normal. The patient responded well to the treatment, tolerated the medication, and had reasonable disease control. Hypoesthesia is the most common treatment-related adverse event (TRAE). There were no grades ≥3 TRAEs. In the subsequent two years of follow-up, there was no disease recurrence.

## Discussion and conclusions

In this case, RC48 monotherapy has demonstrated sound therapeutic effects. Thus, we believe it is appropriate as a first-line therapy for specific patients, such as those unsuitable for cisplatin or those with an eGFR < 45 mL/min.

Despite multiple attempts with new agents and/or combinations of treatments, platinum-based chemotherapy remained the standard of care for previously untreated advanced or metastatic urothelial cancer (1). Systemic platinum-based chemotherapy is effective for both urothelial bladder cancer and UTUC (5), making it the standard treatment for advanced or metastatic UTUC. For patients with an eGFR higher than 45 mL/min, cisplatin-based chemotherapy is widely considered (8). In this specific case, the patient with an eGFR of 69.94 mL/min/1.73 m^2^ underwent systemic chemotherapy with GC and low-dose GC but experienced myelosuppression.

Following this, we transitioned to the second-line immunotherapy with Tislelizumab (1), an anti-human PD-1 monoclonal IgG4 antibody (9). Tislelizumab plus GC significantly improved disease-free survival following RNU and reduced risk by 63.3% compared to the surveillance group, according to Zhang J et al. (p = 0.008) (10). Unfortunately, in this case, the disease continued to progress during immunotherapy. Patients with advanced urothelial carcinoma have poor overall survival (OS) after platinum-based chemotherapy and PD-1 or PD-L1 inhibitor treatment (11).

Ultimately, RC48 was chosen for the patient due to HER2-overexpressing (IHC 3+), leading to disease control and the disappearance of metastatic tumors. RC48 is an ADC consisting of a monoclonal antibody against HER2 conjugated via a cleavable linker to the cytotoxic agent monomethyl auristatin E (7). It reports that the incidence of HER2 overexpression (IHC 2+ or 3+) in urothelial carcinoma ranges from 9.2% to 61.1% (12–14) and 44% in the Chinese population (15). Most of these cases were assessed using IHC or fluorescence *in situ* hybridization. Initially approved for locally advanced or metastatic gastric cancer (7), RC48 is now used for several cancers (16, 17), including metastatic urothelial cancer (18, 19), non-small-cell lung cancer (20), and breast cancer (21). In patients with HER2-positive locally advanced or metastatic urothelial carcinoma that had progressed, Sheng X et al. found that RC48 exhibited a promising efficacy with a tolerable safety profile, with an objective response rate of 50.5%, and median OS and progression-free survival of 5.9 and 14.2 months, respectively (19). According to Xu J et al., eight patients received RC48 monotherapy, while 30 received combination therapy in real-world settings. The median follow-up period was 10.6 months, and progression-free survival rates were 34.1% at 12 months and 63.2% at six months, respectively, with a median and progression-free survival of 8.2 months (95% CI, 5.9-10.5). The average response time was 7.3 months. For metastatic urothelial cancer patients, RC48 showed a controllable safety profile and promising efficacy when used alone or combined with PD-1 inhibitors (22).

In this case, the patient received RC48 monotherapy at 2.5 mg/kg every 14 days for 13 cycles, with positive results. Previous studies mainly involved RC48 in combination therapy, with 2-100 mg/kg dosages for 3-19 cycles (22–26). Therefore, the RC48 monotherapy regimen could be considered a first-line treatment option for selected patients, such as patients unfit for cisplatin or patients with eGFR < 45 mL/min. Our reasoning is as follows: Unlike platinum-based chemotherapy, which requires a certain level of renal function, specifically an eGFR > 45 mL/min, RC48 has been approved without an explicit restriction on eGFR (7); There is literature evidence that RC48 has achieved complete response and long-term progression-free survival (>12 months) in the treatment of metastatic urothelial carcinoma patients with renal insufficiency without worsening of renal function during treatment (27). In a real-world study, RC48 treatment for metastatic urothelial carcinoma patients with a creatinine clearance <30 mL/min had an objective response rate of 40.0%, with no increase in TRAEs in patients with poor condition or impaired renal function (28). Research findings suggest that RC48 treatment can benefit patients with metastatic urothelial carcinoma, irrespective of their poor condition or impaired renal function.

The most common TRAEs associated with RC48 were anemia (71.1%), peripheral sensory neuropathy (68.2%), anorexia (57.9%), asthenia (52.6%), hypoesthesia (52.6%), leukopenia (50.5%), bone marrow suppression (47.4%), and elevated AST levels (42.1%). Immune-related adverse effects were rare (19, 22). In this case, the most frequent TRAE in this case is hypoesthesia. However, the patient was able to tolerate this side effect.

In summary, long-term monotherapy with RC48 is a reasonable and practical option for patients with metastatic disease after RNU, and the side effects are generally manageable for patients. RC48 is suitable as a first-line therapy for selected patients, such as those who are unfit for cisplatin or those with an eGFR < 45 mL/min.

## Data Availability

The original contributions presented in the study are included in the article/**Supplementary Material**, further inquiries can be directed to the corresponding author/s.

## Supplementary material

Supplementary Figure 1 Computed tomography (CT) urography. CT urography shows left hydronephrosis **(A)**, left ureteral dilation **(B)**, a lower ureteral mass **(C)**, and a bladder mass (**D**, as indicated by the arrow).

Supplementary Figure 2 Pathological results of cystoscopy biopsy and surgical specimen. The cystoscopy biopsy results indicate papillary urothelial carcinoma of the bladder, low grade **(A)**. The pathological results of the surgical specimen: (left ureter) non-invasive urothelial carcinoma, low grade, no cancerous tissue seen in the ureteral stump wall **(B)**, gross specimen of the left kidney and ureter **(C)**.

Supplementary Figure 3 Computed tomography (CT) urography and chest CT after three Disitamab vedotin (RC48) treatment cycles. CT urography shows that a mass in the left posterior bladder wall has decreased in size (indicated by the red arrow, **A**). Chest CT shows bilateral pleural effusions, with a large volume on the right side and incomplete expansion of the right lung lobe (indicated by the red arrow, **B**).

## References

[1] Rouprêt M Seisen T Birtle AJ Capoun O Compérat EM Dominguez-Escrig JL. European association of urology guidelines on upper urinary tract urothelial carcinoma: 2023 update. Eur Urol. (2023) 84:49–64. doi: 10.1016/j.eururo.2023.03.013 36967359

[2] Chen XP Xiong GY Li XS Matin SF Garcia M Fang D. Predictive factors for worse pathological outcomes of upper tract urothelial carcinoma: experience from a nationwide high-volume centre in China. BJU Int. (2013) 112:917–24. doi: 10.1111/bju.12238 23905945

[3] Margulis V Shariat SF Matin SF Kamat AM Zigeuner R Kikuchi E. Outcomes of radical nephroureterectomy: a series from the Upper Tract Urothelial Carcinoma Collaboration. Cancer. (2009) 115:1224–33. doi: 10.1002/cncr.24135 19156917

[4] Seisen T Peyronnet B Dominguez-Escrig JL Bruins HM Yuan CY Babjuk M. Oncologic outcomes of kidney-sparing surgery versus radical nephroureterectomy for upper tract urothelial carcinoma: A systematic review by the EAU non-muscle invasive bladder cancer guidelines panel. Eur Urol. (2016) 70:1052–68. doi: 10.1016/j.eururo.2016.07.014 27477528

[5] Alfred Witjes J Max Bruins H Carrión A Cathomas R Compérat E Efstathiou JA. European association of urology guidelines on muscle-invasive and metastatic bladder cancer: summary of the 2023 guidelines. Eur Urol. (2024) 85:17–31. doi: 10.1016/j.eururo.2023.08.016 37858453

[6] Hu Y Chen F Sun S Xv L Wang X Wang M. mTOR inhibitor introduce disitamab vedotin (RC48-ADC) rechallenge microtubule-chemotherapy resistance in HER2-low MBC patients with PI3K mutation. Front Oncol. (2024) 14:1312634. doi: 10.3389/fonc.2024.1312634 38344201 PMC10854197

[7] Deeks ED. Disitamab vedotin: first approval. Drugs. (2021) 81:1929–35. doi: 10.1007/s40265-021-01614-x 34661865

[8] Moschini M Shariat SF Rouprêt M De Santis M Bellmunt J Sternberg CN. Impact of primary tumor location on survival from the european organization for the research and treatment of cancer advanced urothelial cancer studies. J Urol. (2018) 199:1149–57. doi: 10.1016/j.juro.2017.11.068 29158104

[9] Lee A Keam SJ. Tislelizumab: first approval. Drugs. (2020) 80:617–24. doi: 10.1007/s40265-020-01286-z 32185681

[10] Zhang J Yang M Wei D Zhang D Chen Z Zhu H. The efficacy and safety of tislelizumab combined with gemcitabine plus cisplatin in the treatment of postoperative patients with muscle-invasive upper tract urothelial carcinoma. BMC Cancer. (2024) 24:202. doi: 10.1186/s12885-024-11919-1 38350941 PMC10863243

[11] Powles T Rosenberg JE Sonpavde GP Loriot Y Durán I Lee JL. Enfortumab vedotin in previously treated advanced urothelial carcinoma. N Engl J Med. (2021) 384:1125–35. doi: 10.1056/NEJMoa2035807 PMC8450892 33577729

[12] Bellmunt J Werner L Bamias A Fay AP Park RS Riester M. HER2 as a target in invasive urothelial carcinoma. Cancer Med. (2015) 4:844–52. doi: 10.1002/cam4.432 PMC4472207 25720673

[13] Laé M Couturier J Oudard S Radvanyi F Beuzeboc P Vieillefond A. Assessing HER2 gene amplification as a potential target for therapy in invasive urothelial bladder cancer with a standardized methodology: results in 1005 patients. Ann Oncol. (2010) 21:815–9. doi: 10.1093/annonc/mdp488 PMC2844947 19889613

[14] Jimenez RE Hussain M Bianco FJ Jr. Vaishampayan U Tabazcka P Sakr WA. Her-2/neu overexpression in muscle-invasive urothelial carcinoma of the bladder: prognostic significance and comparative analysis in primary and metastatic tumors. Clin Cancer Res. (2001) 7:2440–7. 11489824

[15] Zhou L Shao Z Liu Y Yan X Li J Wu X. HER2 expression associated with clinical characteristics and prognosis of urothelial carcinoma in a chinese population. Oncologist. (2023) 28:e617–24. doi: 10.1093/oncolo/oyad070 PMC10400138 36971495

[16] Hu Y Zhu Y Wei X Tang C Zhang W. Disitamab vedotin, a novel HER2-directed antibody-drug conjugate in gastric cancer and other solid tumors. Drugs Today (Barc). (2022) 58:491–507. doi: 10.1358/dot.2022.58.10.3408812 36305543

[17] Shi F Liu Y Zhou X Shen P Xue R Zhang M. Disitamab vedotin: a novel antibody-drug conjugates for cancer therapy. Drug Delivery. (2022) 29:1335–44. doi: 10.1080/10717544.2022.2069883 PMC9090390 35506447

[18] Padua TC Moschini M Martini A Pederzoli F Nocera L Marandino L. Efficacy and toxicity of antibody-drug conjugates in the treatment of metastatic urothelial cancer: A scoping review. Urol Oncol. (2022) 40:413–23. doi: 10.1016/j.urolonc.2022.07.006 35973928

[19] Sheng X Wang L He Z Shi Y Luo H Han W. Efficacy and safety of disitamab vedotin in patients with human epidermal growth factor receptor 2-positive locally advanced or metastatic urothelial carcinoma: A combined analysis of two phase II clinical trials. J Clin Oncol. (2024) 2023:Jco2202912. doi: 10.1200/JCO.22.02912 PMC11095880 37988648

[20] Ren S Wang J Ying J Mitsudomi T Lee DH Wang Z. Consensus for HER2 alterations testing in non-small-cell lung cancer. ESMO Open. (2022) 7:100395. doi: 10.1016/j.esmoop.2022.100395 35149428 PMC8844658

[21] De Sanctis R Jacobs F Benvenuti C Gaudio M Franceschini R Tancredi R. From seaside to bedside: Current evidence and future perspectives in the treatment of breast cancer using marine compounds. Front Pharmacol. (2022) 13:909566. doi: 10.3389/fphar.2022.909566 36160422 PMC9495264

[22] Xu J Zhang H Zhang L Chu X Li Y Li G. Real-world effectiveness and safety of RC48-ADC alone or in combination with PD-1 inhibitors for patients with locally advanced or metastatic urothelial carcinoma: A multicenter, retrospective clinical study. Cancer Med. (2023) 12:21159–71. doi: 10.1002/cam4.6680 PMC10726858 37935113

[23] Dai L Jin X Wang L Wang H Yan Z Wang G. Efficacy of disitamab vedotin in treating HER2 2+/FISH- gastric cancer. Onco Targets Ther. (2022) 15:267–75. doi: 10.2147/OTT.S349096 PMC8935729 35321517

[24] Wei Y Zhang R Yu C Hong Z Lin L Li T. Disitamab vedotin in combination with immune checkpoint inhibitors for locally and locally advanced bladder urothelial carcinoma: a two-center’s real-world study. Front Pharmacol. (2023) 14:1230395. doi: 10.3389/fphar.2023.1230395 37645442 PMC10461006

[25] Zheng Y Xue YY Zhao YQ Chen Y Li ZP. Disitamab Vedotin plus anti-PD-1 antibody show good efficacy in refractory primary urethral cancer with low HER2 expression: a case report. Front Immunol. (2023) 14:1254812. doi: 10.3389/fimmu.2023.1254812 37901233 PMC10601644

[26] Nie C Xu W Guo Y Gao X Lv H Chen B. Immune checkpoint inhibitors enhanced the antitumor efficacy of disitamab vedotin for patients with HER2-positive or HER2-low advanced or metastatic gastric cancer: a multicenter real-world study. BMC Cancer. (2023) 23:1239. doi: 10.1186/s12885-023-11735-z 38102538 PMC10724908

[27] Xu Z Ma J Chen T Yang Y. Case report: The remarkable response of pembrolizumab combined with RC48 in the third-line treatment of metastatic urothelial carcinoma. Front Immunol. (2022) 13:978266. doi: 10.3389/fimmu.2022.978266 36458005 PMC9705978

[28] Chen J Wang M Qi X Long H Qi N Wu L. RC48-antibody-drug conjugate in metastatic urothelial carcinoma: A multicenter real-world study in China. Clin Genitourin Cancer. (2024) 22:102093. doi: 10.1016/j.clgc.2024.102093 38762350

