# Supplementary material for: Disitamab vedotin (RC48) long-term regimen in a post-nephroureterectomy patient with metastases: a case report
Source: Front Oncol. 2024 Sep 13;14:1419882. doi: 10.3389/fonc.2024.1419882 (PMC11427362; doi:10.3389/fonc.2024.1419882)
Supplement: Supplementary file 1 [file Table1.docx]

|  | Before GC | After GC | Drug discontinuance | After  Low-dose GC | Normal range |
| --- | --- | --- | --- | --- | --- |
| WBC (×10^9^/L) | 3.95 | 1.33 | 7.6 | 2.54 | 3.69-9.16 |
| Neutrophil (×10^9^/L) | 2.57 | 0.81 | 5.61 | 1.36 | 2.00-7.00 |
| RBC (×10^12^/L) | 4.3 | 3.43 | 3.44 | 3.54 | 3.68-5.13 |
| Hemoglobin (g/L) | 131 | 106 | 104 | 109 | 113-151 |
| Platelet (×10^9^/L) | 108 | 22 | 105 | 129 | 101-320 |
| UREA (mmol/L) | 3.2 | 5.2 |  | 5.6 | 2.40-8.20 |
| CRE (μmol/L) | 76 | 84 |  | 77 | 35.0-97.0 |
| AST (IU/L) | 47 | 21 |  | 28 | 0-45 |
| ALT (IU/L) | 44 | 21 |  | 23 | 0-40 |
| eGFR (ml/min/ 1.73 m^2^) | 69.94 | 61.97 |  | 68.37 | ＞90 |
| WBC, White blood cells, RBC, Red blood cells, UREA, Serum urea, CRE, Serum creatinine, ALT, Alanine aminotransferase, AST, Aspartate aminotransferase, eGFR, Estimated glomerular filtration rate, G, gemcitabine, C, cisplatin, | | | | | |

Table 1. Laboratory examinations before and after chemotherapy.
