# Supplementary figures and images for: Disitamab vedotin (RC48) long-term regimen in a post-nephroureterectomy patient with metastases: a case report
Source: Front Oncol. 2024 Sep 13;14:1419882. doi: 10.3389/fonc.2024.1419882 (PMC11427362; doi:10.3389/fonc.2024.1419882)

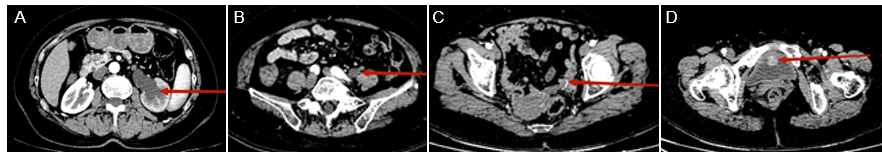

Supplement: Supplementary Figure 1 — Computed tomography (CT) urography. CT urography shows left hydronephrosis (A), left ureteral dilation (B), a lower ureteral mass (C), and a bladder mass (D, as indicated by the arrow). [file Image1.tif]

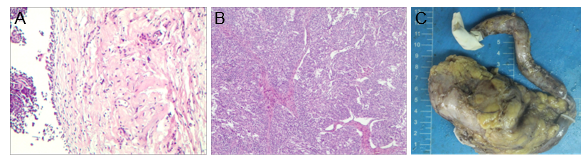

Supplement: Supplementary Figure 2 — Pathological results of cystoscopy biopsy and surgical specimen. The cystoscopy biopsy results indicate papillary urothelial carcinoma of the bladder, low grade (A). The pathological results of the surgical specimen: (left ureter) non-invasive urothelial carcinoma, low grade, no cancerous tissue seen in the ureteral stump wall (B), gross specimen of the left kidney and ureter (C). [file Image2.tif]

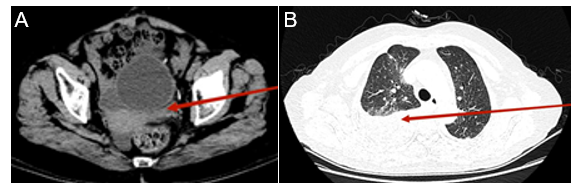

Supplement: Supplementary Figure 3 — Computed tomography (CT) urography and chest CT after three Disitamab vedotin (RC48) treatment cycles. CT urography shows that a mass in the left posterior bladder wall has decreased in size (indicated by the red arrow, A). Chest CT shows bilateral pleural effusions, with a large volume on the right side and incomplete expansion of the right lung lobe (indicated by the red arrow, B). [file Image3.tif]
